# Emerging Trends in Polymer Chemistry and Materials: From Sustainable Design to Advanced Applications

**DOI:** 10.3390/ma19194188

**Published:** 2026-09-30

**Authors:** Gang Wei

**Affiliations:** School of Polymer Science and Engineering, Qingdao University of Science and Technology, Qingdao 266042, China; wei-lab@qust.edu.cn or wei@uni-bremen.de; Tel.: +86-15066242101

## 1. Introduction

Polymeric materials have long transcended their traditional role as structural materials and have become essential to modern technological advancement, profoundly influencing strategic sectors such as biomedicine, energy storage, wearable devices, electronics, environmental science, and advanced space technologies [1,2,3,4,5]. However, the polymer industry has historically relied heavily on non-renewable fossil resources, while the environmental footprint and resource consumption associated with polymer production, use, and end-of-life management have become increasingly challenging [6,7]. Therefore, the sustainable design of polymer-based materials is crucial for promoting their future development, applications, and life-cycle management.

Advanced polymer science is undergoing a profound paradigm shift from traditional performance optimization toward the multidimensional integration of performance, sustainability, intelligence, and manufacturability [8,9]. To enhance material performance, polymers can be combined with functional fillers, including bioactive molecules, functional chemicals, nanoparticles, quantum dots, nanofibers, and two-dimensional (2D) materials [10,11,12], to form polymer–filler composite materials. These functional components can introduce unique physicochemical, biological, optical, electrical, or mechanical properties, thereby expanding the functionality of polymer composites. Surface modification provides another versatile strategy for tailoring properties and interfacial functions of polymers. Physical techniques, including light irradiation, laser treatment, and plasma modification [13,14,15], can be employed to regulate surface chemistry, wettability, adhesion, and other interfacial characteristics.

Sustainability has also become a fundamental consideration in polymer design. The development of recyclable, reusable, and degradable polymer architectures, together with the incorporation of biomass-derived materials and renewable feedstocks, can contribute to more sustainable polymer production and use [16,17]. These approaches can facilitate resource conservation, energy-efficient synthesis, recyclability, and controlled degradation.

Meanwhile, advances in polymer chemistry and materials science are driving the development of adaptive, intelligent, and stimuli-responsive polymeric systems [18,19,20]. Such materials can dynamically adjust their structures and properties in response to external stimuli, including temperature, light, pH, electric or magnetic fields, and mechanical forces. These capabilities provide new opportunities for self-regulation, sensing, actuation, controlled delivery, and precision biomedical applications.

Finally, manufacturability is a critical factor determining the practical translation of polymer materials. Advanced processing strategies enable polymers to be transformed into three-dimensional (3D) hydrogels [21], aerogels [22], foams [23], and other architected structures. Additive manufacturing technologies, particularly 3D and 4D printing [24,25], further enable the fabrication of programmable 3D materials and devices with precisely controlled architectures and functions. Collectively, these advances are reshaping polymer science from a property-centered discipline toward an integrated platform for designing sustainable, intelligent, multifunctional, and manufacturable materials.

## 2. Overview of the Editor’s Choice Articles

In this collection, 15 articles, including 3 reviews and 12 research articles, were selected as the 2025 Best Papers of the Polymeric Materials Section of this journal by the Editorial Board Members. This collection also places particular emphasis on the importance of interdisciplinary collaboration and synergistic innovation. These cutting-edge advances rely on the deep integration of polymer synthesis and chemistry, computational materials science, nanotechnology, bioengineering, and advanced manufacturing technologies.

### 2.1. Sustainability of Polymers

Sustainability has become a central theme in modern polymer science, driven by the need to reduce resource consumption, waste generation, and the environmental impact of polymeric materials. Various sustainable strategies, such as bio-based polymers, chemical and mechanical recycling, biodegradable and compostable polymers, and recyclable polymer systems have been widely utilized in recent years [26,27]. Additionally, sustainable polymers have also been integrated with green synthesis, life-cycle evaluation, and circular-economy principles to enhance or balance material performance with environmental responsibility [28,29].

Several contributions from this collection highlighted recent approaches toward sustainable polymer development and circular material use. In a review [30], Gadaleta and co-workers presented a comprehensive review on the regulation of the biodegradability of bioplastics under laboratory and real-world environments, including composting and anaerobic digestion, soil, freshwater, and marine systems. They presented the biodegradation mechanisms of various bioplastics that fabricated with polylactic acid (PLA), polycaprolactone (PCL), starches, cellulose, and others, and introduced and discussed the methods for assessing the biodegradability of these bioplastics. By highlighting the dependence of degradation on both material characteristics and environmental factors, the review provides useful guidance for polymer material selection, environmental risk assessment, and sustainable polymer design. Such an approach could help reconcile durability during use with controlled degradation after disposal and ultimately support more credible circular and sustainable polymer systems. To achieve the sustainable utilization of polypropylene (PP) plastic materials, Knoben et al. modified recycled PP with an antioxidant (Irganox 1010) and then measured the values of oxidation induction temperature [31]. They addressed an important challenge in the mechanical recycling of PP by focusing on the evolution of antioxidants during repeated processing. Additionally, the antioxidant consumption and accumulation under open-loop and closed-loop recycling scenarios were analyzed. Understanding antioxidant depletion and build-up provide a scientific basis for determining when and how stabilizers should be replenished during the recycle of polymers.

To present sustainable applications of biomass polymers, Macena et al. characterized the chemical composition, structural characteristics, and physicochemical properties of lignocellulosic byproducts form the Portuguese Forest with multiple techniques [32]. Such characterizations are important as lignocellulosic wastes are heterogeneous, and their composition strongly determines their suitability for extraction, bioenergy, biocomposites, packaging, and other value-added products. Their study proves that the transfer of forest biomass byproducts into functional resources can reduce waste, decrease dependence on fossil-derived materials, and create additional value from existing forestry systems, endowing high sustainability for biomass. In addition, the functionalization of polymers with natural biomaterials can improve their sustainable applications. For instance, Fabijański and co-workers presented a sustainable approach to modifying PLA through the incorporation of powdered cork, combining a biodegradable polymer matrix with a renewable and underutilized natural filler [33]. As a functional biomaterial-based reinforcement, the integration of powdered cork with PLA altered its mechanical, thermal, and processing characteristics while increasing the renewable content of the resulting composite materials. This study provides a pathway toward more sustainable polymer composites with reduced dependence on conventional inorganic fillers. Furthermore, the work contributes to the development of bio-based composite materials within a circular-economy framework.

### 2.2. Design and Synthesis of Polymer Composites and Nanocomposites

Polymer composites and nanocomposites provide effective strategies for tailoring mechanical, chemical, thermal, and functional properties beyond those of conventional polymers. The design process typically considers the polymeric matrix type, reinforcement composition, particle size and morphology, interfacial interactions, dispersion, and processing conditions [34,35]. Various additives such as nanoparticles, nanofibers, 2D materials, and others have widely used to integrate with polymers to form polymer nanocomposites previously [36,37]. Recently, more and more attention has been attracted on matrix–filler interfaces and filler dispersion, renewable reinforcements, recycled polymers, low-energy processing, and recyclable composite architectures [38,39].

Wakeel et al. provides a valuable overview of the development of polymer nanocomposites for advanced drilling-fluid systems, highlighting how nanoscale fillers can enhance the rheological, filtration, thermal, and mechanical stability of conventional polymer-based fluids [40]. It brings recent progress on polymer–nanoparticle interactions, nanocomposite design, and multifunctional drilling-fluid performance, thereby connecting polymer science with the practical requirements of complex drilling environments. In addition, it can promote the development of more efficient and reliable drilling technologies while potentially reducing fluid consumption and environmental impacts. In another review [41], Janeikaite and co-workers summarized recent advances in the construction of fiber-reinforced polymer (FRP) laminates for aviation and structural engineering, with particular emphasis on performance requirements, design principles, and defect assessment. It connected two application fields that have different safety requirements, loading conditions, manufacturing practices, and inspection standards, while identifying common principles governing FRP laminate design and failure, providing a useful framework for engineers selecting and managing composite laminates across different structural applications.

The functionalization of polymers with inorganic nanoparticles offers an effective strategy for tailoring material properties, including mechanical, electrical, thermal, optical, and biological functions for extended applications [42]. Nakagawa and Iritani reported the modification of resin with ZnO particles for developing ZnO-reinforced resin composites through supramolecular interactions [43]. Using the powerful surface characteristics of ZnO, they established noncovalent interactions with the resin matrix, providing an alternative to conventional covalent modification or simple physical blending. Such supramolecular interactions improved ZnO dispersion and interfacial adhesion while potentially retaining the intrinsic optical, thermal, and functional properties of ZnO, achieving simultaneous improvements in mechanical performance and functions of polymer composites. In another study, Magiera et al. modified polyurethane (PU) foam with coal processing-derived fillers from waste [44]. They examined the effects of waste incorporation on the structural, chemical, and mechanical characteristics of PU foam and provided insight into how waste-derived components influence the resulting polymer composites. Their results indicated that the addition of waste fillers did not change the cellular and chemical composition of PU, but rather accelerated the functions of aging and thermal stability. The combined structural and mechanical characterization also helps establish the feasibility of converting thermal-processing waste into functional polymeric materials.

### 2.3. Advanced Construction Techniques of Smart Polymer Materials

The advanced construction of smart polymer materials focuses on creating intelligent polymers and polymer-based architectures with precisely controlled molecular structures, hierarchical organization, and multifunctional properties [45]. By manipulating their molecular structure, sequence, crosslinking, supramolecular interactions, and nanoscale/microscale organization, polymers can be engineered to exhibit properties beyond those of conventional materials [46,47,48]. In addition, it is possible to enable the polymer materials with multiple functions through advanced construction like 3D/4D printing and electrospinning, showing unique properties such as mechanical robustness, stimuli-responsiveness, self-healing, and bioactivity [49,50].

Vanja et al. evaluated the effects of seawater and sunlight on the aging-induced mechanical performance of knitted swimwear fabrics [51]. Their result proved that aging altered the dependence of mechanical properties on fabric; for instance, seawater enhanced the strength in the wale direction and sunlight shifted the effects toward the strength in the course direction. To reduce the effects of aging on polymer materials, Martínez and co-workers attempted the plasma treatment of rubber to reduce the aging effect [52]. The coating adhesion and tensile performance of rubber before and after aging were measured. Their results indicated plasma treatment modified the rubber surface chemistry and wettability without substantially altering the bulk material, thereby providing an attractive route for improving coating–rubber interfacial bonding. In another study, Xie and co-workers studied the thermo-oxidative aging effects on ethylene propylene diene monomer (EPDM) rubber by combining experimental characterization with constitutive modeling [53]. They established a quantitative relationship between aging-induced changes in the hyperelastic response and the parameters of constitutive models, providing a useful framework for predicting material performance and supporting a durability-oriented design.

Additive manufacturing is opening new opportunities for the fabrication of polymer materials with complex architectures and application-specific properties. For instance, Poplawski et al. presented a valuable approach for understanding the mechanical behavior of photopolymer resin lattice structures fabricated by additive manufacturing by integrating digital image correlation (DIC) with numerical analysis [54]. It is significant for the rational design and optimization of lightweight, architected polymer structures for biomedical, aerospace, and engineering applications. In another study, Snarski-Adamski et al. produced orthodontic devices with 3D digital light processing (DLP) technology and evaluated the tribological behavior of printed devices [55]. The friction, wear, hardness, stiffness, scratch resistance, and surface characteristics under controlled temperature and humidity conditions of the printed polymer materials were analyzed. Additionally, the comparison of commercially available resins also provides useful practical guidance for polymer material selection and device fabrication.

### 2.4. Biomedical Applications of Polymer Materials

Polymer materials are also playing an increasingly important role in biomedical engineering, particularly through the development of polymer-based hydrogels and functional nanomaterials [56]. Various natural and artificial polymer materials from collagen, chitosan, hyaluronic acid (HA), PLA, PLGA, PCL, and PEG have been constructed. Their tunable biocompatibility, biodegradability, mechanical properties, and functionality enable applications in drug delivery, tissue engineering, wound healing, implants, biosensors, and regenerative medicine [57,58,59,60].

Yoo et al. reported the light-intensity-dependent control of collagen hydrogel properties through photo-crosslinking mediated with riboflavin phosphate (RFP) [61]. In their study, RFP was utilized to regulate the crosslinking process and consequently control the structural, mechanical, and physicochemical properties of collagen hydrogels. Compared with conventional chemical crosslinking approaches, this method offers spatiotemporal control, mild processing conditions, and potentially improved compatibility with biological systems, showing their promising applications in tissue engineering and regenerative medicine.

In another study, Chandrasekaran and co-workers reported the synthesis of HA-derived carbon quantum dots (CQDs) for the therapy of triple-negative breast cancer (TNBC) cells [62]. As HA exhibits potential targeting for CD44 receptors, the thermally synthesized HA-CQDs achieved tumor cell targeting and ferroptosis induction, enabling selective interaction with CD44-overexpressing TNBC cells while promoting ferroptotic cell death. Additionally, their results indicated that the antitumor mechanism of HA-CQDs are related to the pathway of lipid peroxidation induced by reactive oxygen species (ROS) and triggering ferroptosis via downregulation of glutathione and SLC7A11. This study is helpful for engineering polymer-based multifunctional nanomaterials for targeted cancer therapy.

## 3. Challenges and Future Directions

### 3.1. Challenges

Although great achievements have been obtained, polymer chemistry and materials science face several interconnected challenges in developing next-generation polymeric systems. First, precise polymer molecular design requires accurate control over molecular weight, chain architecture, sequence, stereochemistry, and functional groups to achieve predictable properties and functions. Second, establishing structure–property relationships is hard because polymer performance is governed by complex interactions across molecular, nanoscale, and macroscopic length scales. Advanced characterization, computational modeling, and artificial intelligence (AI) analysis are required to develop more predictive design strategies. Third, dynamic functionality of polymer networks is always challenging to create adaptive, self-healing, stimuli-responsive, and intelligent materials. A major challenge is balancing responsiveness and reversibility with mechanical strength, stability, and durability. Fourth, sustainability requires reducing dependence on fossil resources and developing polymers with good biocompatibility, biodegradation, stability, and bioactivity. Finally, it is also challenging to translate laboratory products into real-world applications, as large-scale production may affect material structure and performance.

### 3.2. Future Directions

Future directions of polymer chemistry and materials could be focused more on molecular design, precise polymer functionalization, advanced fabrication, and industrial applications.

First, polymer molecular design provides a fundamental basis and an important starting point for the development of functional polymer materials. It involves the deliberate control of polymer molecular structures to achieve targeted properties and functions by tailoring parameters such as monomer structure, molecular weight, chain architecture, sequence, branching, and functional groups. These structural features can be regulated through strategies including controlled polymerization, copolymerization, and post-polymerization modification. Representative advances include side-chain engineering and chemical doping of conjugated polymers [63], as well as the design of mechanically interlocked 2D polymers [64], which demonstrate how molecular-level structural control can generate novel properties and functions.

Second, chemical upcycling and dynamic covalent networks of polymers should be carried out. It is a key topic to developing reprocessable polymer networks through the incorporation of dynamic covalent bonds such as ester exchange, disulfide, and imine bonds [65]. For instance, Wu et al. designed a dynamic crosslinked topological polymer network that can address the trade-off between high-tensile properties and processability of polymer materials [66]. In addition, a triple dynamic polymer network mediated the creation of epoxy vitrimer by carbon fiber-reinforced composites, which exhibited good self-healing, reusable degradation, and close-loop recycling [67]. In addition, catalytic depolymerization can be used to convert waste plastics, such as PET and polyurethane, directly into high-value fine chemicals or pharmaceutical intermediates [68].

Third, the sustainability of polymer materials is still the main aim. The design and synthesis of natural biomass (lignin, cellulose, chitosan, and others) and synthetic polymer derivatives with good biodegradability, high performance, and closed-loop recyclable capacity are useful in achieving this aim. The using of biomass molecules [69], bioinspired polymers [57], and organic porous polymers (MOFs/COFs) [70] for the synthesis of multifunctional polymer materials showed high sustainability for different applications.

Fourth, additive manufacturing and 3D/4D printing techniques are highly recommended to fabricate polymeric shape-memory materials, stimuli-responsive materials, wearable sensors, and other intelligent structures with programmable properties. These constructed hydrogel and aerogel materials and devices could be potentially utilized for space exploration, disease monitoring, biomedicine, and soft robotics.

Fifth, machine learning-medicated polymer synthesis and structure–property prediction will promote the quick development of polymer chemistry and materials. Powerful AI algorithms are beneficial to predict various polymerization conditions and product properties, which can shorten the materials development cycle and accelerate the discovery of advanced functional polymer materials. In a recent study, a molecularly imprinted polymer sensor was integrated with deep learning, promoting the establishment of a next-generation electrochemical biosensor for monitoring oral inflammation [71]. Furthermore, another study using machine learning and big data mining demonstrated the predictable separation of CO_2_ with polymer–MOF composite membranes [72].

## 4. Conclusions

In summary, this collection moves beyond the conventional linear paradigm of materials synthesis and focuses more on how the principles of green chemistry, advanced functional design, and digital manufacturing technologies can be integrated throughout the entire life cycle of polymeric materials. The contributions collected in this issue cover several compelling and emerging frontiers. From the aspect of sustainability, researchers are developing bio-based and renewable polymer monomers, dynamic covalent crosslinked networks, and efficient chemical recycling systems, with the aim of addressing the long-standing trade-off between environmental sustainability and material performance. Due to their functionality and intelligence, the precise regulation of polymer–filler interfacial interactions, incorporation of stimuli-responsive components, and construction of hierarchically ordered structures have substantially enhanced the mechanical, thermal, and electrical performance of polymer composites. In addition, smart and stimuli-responsive polymers precisely targeted biomedical hydrogels, and structure–function integrated devices enabled by photopolymerization-based additive manufacturing were also presented. These advances are accelerating the translation of laboratory innovations into practical applications, including clinical diagnosis and therapy, flexible sensing, and lightweight engineering systems.
